# Fishing for drifts: detecting buoyancy changes of a top marine predator using a step-wise filtering method

**DOI:** 10.1242/jeb.118109

**Published:** 2015-12

**Authors:** Samantha Alex Gordine, Michael Fedak, Lars Boehme

**Affiliations:** Sea Mammal Research Unit, Scottish Oceans Institute, University of St Andrews, St Andrews, Fife KY16 8LB, UK

**Keywords:** Body condition, Marine mammal, Elephant seal, Body composition, Drift diving, Telemetry, Foraging ecology, Diving behaviour

## Abstract

In southern elephant seals (*Mirounga leonina*), fasting- and foraging-related fluctuations in body composition are reflected by buoyancy changes. Such buoyancy changes can be monitored by measuring changes in the rate at which a seal drifts passively through the water column, i.e. when all active swimming motion ceases. Here, we present an improved knowledge-based method for detecting buoyancy changes from compressed and abstracted dive profiles received through telemetry. By step-wise filtering of the dive data, the developed algorithm identifies fragments of dives that correspond to times when animals drift. In the dive records of 11 southern elephant seals from South Georgia, this filtering method identified 0.8–2.2% of all dives as drift dives, indicating large individual variation in drift diving behaviour. The obtained drift rate time series exhibit that, at the beginning of each migration, all individuals were strongly negatively buoyant. Over the following 75–150 days, the buoyancy of all individuals peaked close to or at neutral buoyancy, indicative of a seal's foraging success. Independent verification with visually inspected detailed high-resolution dive data confirmed that this method is capable of reliably detecting buoyancy changes in the dive records of drift diving species using abstracted data. This also affirms that abstracted dive profiles convey the geometric shape of drift dives in sufficient detail for them to be identified. Further, it suggests that, using this step-wise filtering method, buoyancy changes could be detected even in old datasets with compressed dive information, for which conventional drift dive classification previously failed.

## Abstract (precis)

**Summary:** A step-wise filtering method to detect buoyancy changes in drift diving pinnipeds.

## INTRODUCTION

For marine divers, buoyancy is incredibly important for maintaining position at depth and buoyancy changes have consequences for the energetics of swimming. Changes in an individual's buoyancy, and hence changes in the costs of locomotion, are likely to result in behavioural adjustments that re-establish cost-efficient locomotion and effective foraging. For example, some species actively manipulate their buoyancy by changing the air volume in their lungs or pelage (Kooyman, 1973; Lovvorn and Jones, 1991; Minamikawa et al., 1997), while others adapt their diving behaviour to optimally use their own buoyancy (Beck et al., 2000; Elliott et al., 2007; Watanabe et al., 2006; Webb et al., 1998).

However, buoyancy is also determined by body composition (Beck et al., 2000; Biuw et al., 2003; Lovvorn and Jones, 1991; Webb et al., 1998), which changes markedly because fasting, growth and/or successful foraging alter the ratio of high-density lean and low-density lipid tissue (Beck et al., 2000; Biuw et al., 2003; Fedak et al., 1994; Lovvorn and Jones, 1991; Webb et al., 1998). Detecting buoyancy changes thus can provide crucial information for monitoring the well-being of far-ranging marine mammals such as southern elephant seals (*Mirounga leonina* Linnaeus 1758) and for investigating buoyancy-related changes in their diving behaviour.

Elephant seals spend most of their life at sea, diving for about 90% of the time once they leave land after breeding or moulting (Biuw et al., 2003; Costa, 1993). They regularly perform dives containing inactive phases during which they are thought to rest or sleep whilst passively drifting to depths of 550 m. Such dives, during which the animal stops swimming actively and drifts passively in the water column, are known as drift dives. The rate at which an individual drifts up or down in the water column reflects its buoyancy (Webb et al., 1998). Identifying drift dives and examining drift rate changes thus enables us to monitor buoyancy changes and to indirectly measure changes in body composition and successful resource acquisition (Biuw et al., 2003, 2007; Crocker et al., 1997).

Various methodologies for drift dive identification rely on visual dive classification (Hassrick et al., 2007; Kuhn et al., 2009; Le Boeuf et al., 1992; Page et al., 2005), the use of statistics-based identification algorithms (Miller et al., 2012; Robinson et al., 2007; Thums et al., 2008a) or the application of knowledge-based selection criteria (Biuw et al., 2003; Guinet et al., 2014; Mitani et al., 2010). All three approaches present operational difficulties. Visual dive classification is time consuming and subjective; the implementation of automated search algorithms is to some degree subjective and requires knowledge of the underlying statistical programming; existing knowledge-based methods – all based on Biuw et al. (2003) – have not been revised or modified in light of current knowledge of drift diving behaviour or the frequent implementation of the broken-stick model for dive abstraction (Fedak et al., 2002; Photopoulou et al., 2015).

Advances in telemetry, in particular the use of accelerometers to monitor thrusting and attitude during swimming, improve drift dive identification. However, to obtain these data, either high-bandwidth data transmission or recovery of instruments with large memories is required – both of which provide obstacles in studying animals that only infrequently surface or return to shore. Therefore, data compression and abstraction currently remains the only viable alternative for animal-borne telemetry.

Here, we present a new method based on filtering broken-stick abstracted dive data (Fedak et al., 2001) for detecting buoyancy changes. By carefully considering current knowledge of drift diving behaviour, previously published knowledge-based criteria were revised and new selection criteria were devised to inform this filtering method. We used a combination of telemetered and recorded dive data to test and verify this step-wise filtering method. By testing the method on abstracted and compressed telemetry dive data attained via the low bandwidth Argos System (Argos, 2011), drift rate information was obtained for 11 southern elephant seals from South Georgia, South Atlantic. For five southern elephant seals from the Kerguelen Islands, the results of applying the step-wise filtering method were verified with visual inspection of additional recorded high-resolution dive data.

## MATERIALS AND METHODS

### Logger deployments

Satellite-relay data loggers [SRDLs; Sea Mammal Research Unit (SMRU) Instrumentation, St Andrews, UK] were deployed on 12 southern elephant seals on the island of South Georgia, South Atlantic, in the Austral summer of 2009 as part of the SAVEX project (http://www.st-andrews.ac.uk/~savex) and the data from 11 seals were used for further analysis. Additionally, five seals were equipped with fluorometry-conductivity–temperature–depth satellite-relay data loggers (Fluoro-CTD-SRDLs; SMRU Instrumentation) on the Kerguelen Islands in the Austral summers of 2011 and 2012 and in the Austral winter of 2012 as part of the MEMO Observatory (http://www.insu.cnrs.fr/node/4125).

The capture and tagging protocols of the UK deployments on South Georgia were reviewed and approved by the University Teaching and Research Ethics Committee (UTREC) and the Animal Welfare and Ethics Committee (AWEC) as part of our ethical review process and were scrutinised under the UK Animals (Scientific Procedures) Act 1986. Capture and deployment of satellite transmitters were carried out by experienced personnel with UK Animals (Scientific Procedures) Act 1986 Personal Licences. The capture and tagging of the French deployments on the Kerguelen Islands were undertaken with approval from the IPEV (Institut Polaire Français Paul Emile Victor) and TAAF (Terres Australes et Antarctiques Francaises) animal ethics committee.

The SRDLs recorded high-resolution time–depth profiles at 2 s intervals. These detailed time–depth profiles were then abstracted using a broken-stick algorithm on-board the SRDLs which reduced the data to four at-depth and two surface dive inflection points (Fedak et al., 2001; Photopolou, 2012). The abstracted data were then compressed and relayed via the Argos satellite system (Argos, 2011; Boehme et al., 2009; Fedak et al., 2002). Additionally, the detailed high-resolution data stored on-board the Fluoro-CTD-SRDLs of the five Kerguelen seals were recovered upon recapturing the animals.

### Data handling

Below, we present a filtering method that automatically searched through all abstracted time–depth profiles to identify dive profiles that included a drift fragment. To see whether the rate at which the individual drifts changed over time, the drift rates of all identified drift fragments were extracted as a time series. For seals from South Georgia, these drift rate time series were further investigated with regard to drift dive characteristics, behavioural changes and emergent buoyancy changes. The drift rate time series of the five southern elephant seals from the Kerguelen Islands were solely used for the independent verification of the developed step-wise filtering method. The relevant data analysis was carried out using the programming software R (R-Core-Team, 2013), unless stated otherwise.

### Data preparation for step-wise filtering

**Fig. 1.**
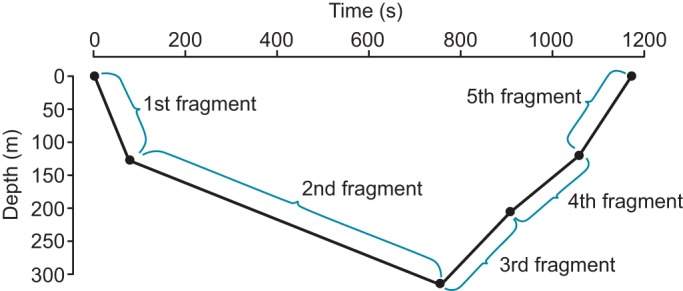
**A reconstructed broken-stick abstracted time–depth profile of a typical drift dive.** The black circles indicate the two at-surface and four at-depth inflection points chosen by the broken-stick algorithm on-board the satellite-relay data loggers (SRDLs). The segment between two successive inflection points describes each dive fragment, with five fragments per dive profile in total. In this example, the second dive fragment is the drift fragment of this dive profile.

Dive profiles with dive duration recorded as zero were removed. Each dive profile was subdivided into five dive fragments (Fig. 1) using the six inflection points provided by the broken-stick algorithm. Dive fragments described by two consecutive inflection points of the same recorded depth were excluded to prevent flat-bottomed dives from being misidentified as drift dives. The first and last dive fragments of each profile, representing descents and ascents, were excluded.

The vertical speed of each fragment was calculated by dividing the difference in depth by the difference in time of the two inflection points describing the fragment. All speeds referred to in this study are vertical speeds, with an expected error of <5%, based on the accuracy of the SRDL sensors. The percentage duration of each dive fragment was calculated by dividing the duration of a given fragment by the total dive duration. Tags were collecting data using GMT (Greenwich Mean Time) and local time (lt) was calculated based on the specific longitude at that time.

### Step-wise filtering method using selection criteria

The automated detection of drift dives previously used criteria such as the proportional length of a drift fragment to identify drift dives (Biuw et al., 2003; Mitani et al., 2010; Onoufriou, 2012). Recent advances in drift diving analysis enabled the revision of existing selection criteria in addition to the formulation of new selection criteria to refine drift dive analysis. The step-wise application of the seven selection criteria outlined below discarded dive fragments that did not fulfil all the chosen selection criteria. Dive fragments that remained after filtering were considered to be drift fragments and their vertical speeds represent drift rates.

The selection criteria used by the step-wise filtering method were as follows. (1) Only recently weaned elephant seal pups (Biuw et al., 2003) or females during late gestation (Crocker et al., 1997) are known to become positively buoyant. *A priori*, we do not expect seals to reach positive buoyancy. Based on this and the minimum drift rates found by Bailleul et al. (2007), fragments with vertical speeds between −0.05 and −0.6 m s^−1^ (Dragon et al., 2012; Onoufriou, 2012) were thus retained.

**Fig. 2.**
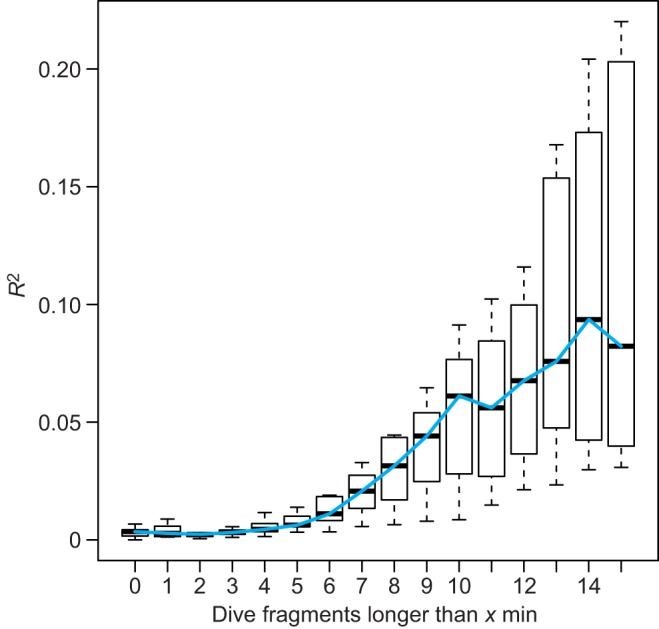
**Boxplot of coefficients of determination (*R***^**2**^**) for determining the minimum length of a drift fragment.** Smooth splines were fitted to the time traces of vertical speeds using the data in each subsample (see Materials and methods). The dive fragments in each subsample were longer than a pre-determined length (*x* min) and were derived from one of the 11 individuals. For each fitted spline, *R*^2^ was calculated as *R*^2^=1−(residual sum of squares/total sum of squares). This boxplot summarises the splines' goodness of fit. The fit improves starkly for splines fitted to vertical speed time series of fragments longer than 8 min.

(2) Elephant seals typically begin to drift at depths between 65 and 117 m (Biuw et al., 2003), with 95% of all drifts occurring below 100 m (Bailleul et al., 2007). Critical evaluation of the maximum depth of drift dives (Aoki et al., 2011; Crocker et al., 1997) attests that the majority of drifts are terminated at depths shallower than 550 m. Shallow dives, during which residual air in the lungs has a variable influence on buoyancy, were excluded (Biuw et al., 2003). The buoyant force of residual air becomes negligible below a depth of 100 m because the lungs of elephant seals are then collapsed by the surrounding pressure (Kooyman and Ponganis, 1998). Therefore, only dive fragments starting and ending in the depth range of 100–550 m were retained.

(3) Varying information is available on the length of a drift fragment. Crocker et al. (1997) recorded mean lengths of approximately 12 min for early gestation and approximately 13 min for late gestation female elephant seals. Aoki et al. (2011) found that, on average, drift fragments lasted for approximately 7 min. To determine a minimum drift length, we therefore chose a statistics-based approach. (i) The data of each individual were divided into subsamples according to the length of dive fragments. Thus, the data in each subsample only contained dive fragments that were longer than a certain fragment length, e.g. longer than 1 min, or 2 min, or 3 min, etc. (ii) The vertical speeds of the dive fragments in each subsample were plotted against time (see Fig. S1). (iii) The smooth.spline function (Venables and Ripley, 1994), as implemented in the R package (Ihaka and Gentleman, 1996), was fitted to these time series of vertical speeds for each subsample (see Fig. S1). (iv) For these splines, the coefficients of determination (*R*^2^) were calculated (Fig. 2) to assess the fit of the spline to the raw data points of each subsample. (v) The *R*^2^ values of all splines from all individuals were examined for the most substantial improvement of fit, i.e. the inflection point (Fig. 2). The first inflection point was obtained for splines fitted to dive fragments longer than 8 min, whilst the first maximum was attained for dive fragments longer than 10 min (Fig. 2). Overall, the longer the dive fragments, the better the fit of the splines. However, there was also a trade-off between improvement of fit and the number of dive fragments that were retained. When the other six selection criteria were applied, dive fragments longer than 8 min were most frequent (see Fig. S2). Considering this, the minimum fragment length was chosen to be greater than 8 min.

(4) The purpose of a drift dive is to rest or process food whilst drifting passively. Thus, the drift fragment should consume most of the dive time. Therefore, all potential drift fragments shorter than 40% of the total dive duration were discarded (Andersen et al., 2014; Biuw et al., 2003; Onoufriou, 2012).

(5) After passively drifting at depth for extended periods, seals must surface again to breathe. Therefore, the dive fragment after the potential drift fragment should have a vertical speed that is indicative of active, upward swimming. Thus, dive fragments with subsequent fragments below 0.2 m s^−1^ were discarded (Richard et al., 2014).

(6) Drift dives occur more often during the night (Andersen et al., 2014; Dragon et al., 2012; Le Boeuf et al., 2000). Elephant seals drift dive most frequently between 05:00 lt and 07:00 lt in the morning, and least often between 14:00 lt and 19:00 lt (Crocker et al., 1997). Thus, taking a conservative approach, dive fragments occurring between 13:30 lt and 19:30 lt were discarded.

(7) An elephant seal swims actively upon descent before beginning its drift. The vertical speed of the dive fragment preceding the potential drift fragment should reflect this. Thus, dive fragments with preceding fragments above −0.6 m s^−1^ were discarded.

### Spline fitting

We recommend fitting a spline or regression line to the drift rate time series to study the trend in buoyancy change and to estimate changes in drift rate by predicting representative daily values.

A range of spline-fitting algorithms and non-parametric regression methods [smooth.spline (Venables and Ripley, 1994); loess (Cleveland et al., 1992); gam (Hastie, 1991)] were tested by visually inspecting which splines fitted the trend in individual drift rate time series best and which spline-generating method most frequently provided the closest fit overall without changing fitting parameters for each time series. Subsequently, we chose constrained beta splines (cobs) (Ng and Maechler, 2007), as these splines consistently provided the best fit.

For the cobs algorithms, the constraint ‘none’ was chosen, because time series of single migrations do not provide enough data to demonstrate periodicity. The maximum number of knots was set to 14 to prevent over-fitting and to permit any biologically meaningful changes in drift rate presumable over a week to be detected (Biuw et al., 2003). Knots were generated using the quantile method and the desired quantile level that provided the best results was 20%. Penalty parameters did not improve the fit. When dive records continued to be recorded after an extended haul-out period, separate splines were generated for the pre- and post-haul-out periods.

### Independent verification

The step-wise filtering method was applied to the broken-stick abstracted time–depth profiles of the five southern elephant seals from the Kerguelen Islands, which were received by telemetry. The detailed high-resolution time–depth profiles (sampled at 2 s intervals) of these broken-stick abstracted dive profiles were visually inspected for drift dives, using MAMVIS (Fedak et al., 1996). Drift dives undetected by the step-wise filtering method and dives falsely identified as drift dives by the filtering method were counted. The drift rates of correctly identified drift dives were re-measured by inspecting the relevant drift phase of the detailed high-resolution dive profile in MAMVIS.

A statistical algorithm adapted from Dragon et al. (2012) was run using MATLAB (The MathWorks Inc., 2011) to create a detailed drift rate time series for each animal. For this, the detailed time–depth data recorded at 2 s intervals were down-sampled to 40 s interval data. The mean vertical speed and standard deviation within an 8 min sliding window were calculated for data below a depth of 50 m. Drift phases were detected as phases with a low absolute mean vertical speed (<1 m s^−1^) and a low standard deviation (<0.05 m s^−1^ for seals ft12-F1-12, ft12-F4-12 and ft07-cy28-11; <0.075 m s^−1^ for seal ft12-F2-12; <0.1 m s^−1^ for seal ft11-cy30b-12).

### Time series comparison

To reduce the high daily variation in drift rate, the mean daily drift rate was computed for each time series.

The drift rate time series are non-stationary because the drift rates in each time series are strongly auto-correlated. To ensure that the correlation between the visually confirmed and the filtered time series was not an artefact of auto-correlation, autoregressive integrated moving average (ARIMA) models were fitted to each daily mean drift rate time series using automated parameter selection (Hyndman and Khandakar, 2008). ARIMA models forecast time series that are made stationary by differencing so that the time series’ autocorrelations remain constant over time (Pfaff, 2008).

The time series were differenced one lag at a time by as many differencing steps as the automatic ARIMA parameter selection suggested were necessary to make the time series stationary (Pfaff, 2008). For each individual, this rendered a set of two forecasted time series: one based on the filtered drift rate time series and a corresponding one based on the visually inspected drift rate time series. Within each set, the corresponding difference-stationary time series were statistically compared using Pearson's product moment correlation test (Table 1).

**Table 1.**
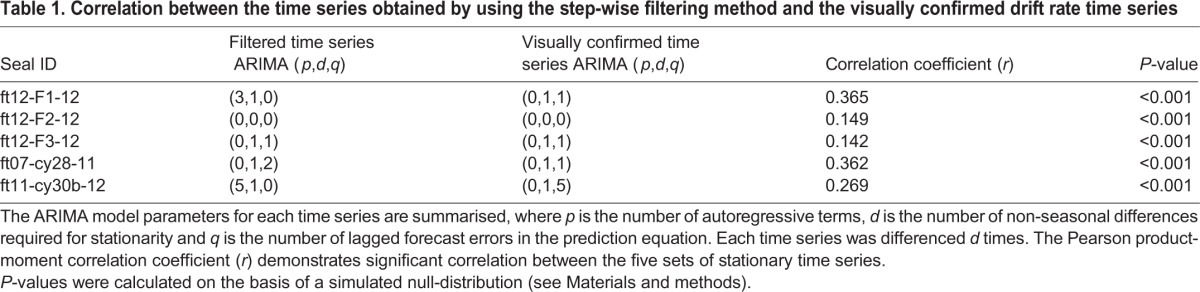
**Correlation between the time series obtained by using the step-wise filtering method and the visually confirmed drift rate time series**

Further, a null-correlation distribution was generated by using the automatically selected ARIMA model parameters to simulate sets of time series with the same known autocorrelation structure. For each individual, this rendered a set comprising 1000 simulated time series based on the model parameters of the filtered time series and 1000 simulated time series based on the model parameters of the corresponding visually confirmed time series. Again, within each set, the simulated time series were compared using Pearson's product moment correlation test, generating a null-distribution of correlation coefficients. This distribution was then used to calculate the probability of obtaining the observed correlation coefficient by chance (*P*-value) (Table 1).

## RESULTS

The dive records collected by the 11 seals from South Georgia covered several months so that sufficiently long drift rate time series could be obtained for all individuals. The average trip duration was 212±87 days (mean±s.d.). A total of 59,240 complete dive profiles with five dive fragments each were considered. After filtering, 2322 dive fragments remained that met all chosen selection criteria. This corresponds to 0.8–2.2% of an individual's dive fragments being selected as drift fragments by the filtering method (Table 2).

**Table 2.**
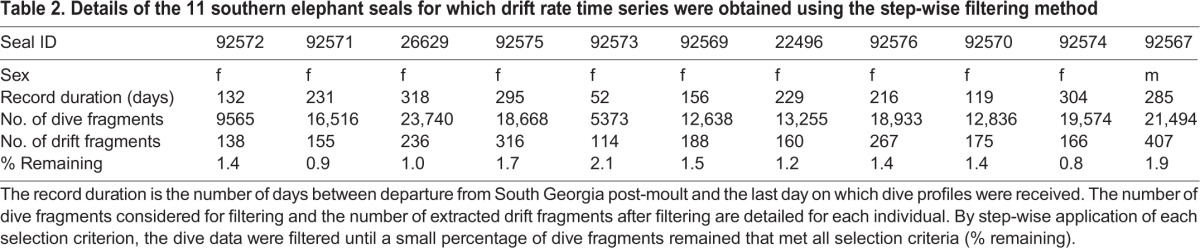
**Details of the 11 southern elephant seals for which drift rate time series were obtained using the step-wise filtering method**

**Fig. 3.**
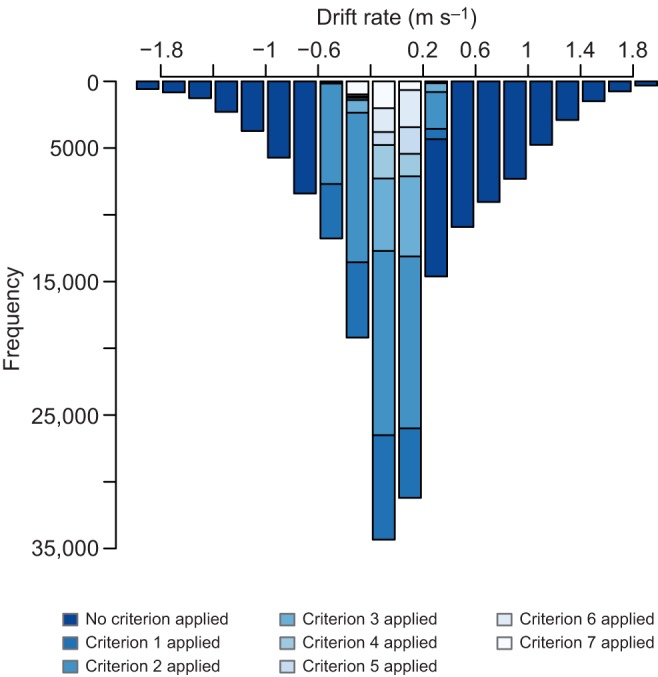
**Histogram of vertical speed of dive fragments that remained after step-wise application of selection criteria.** Step-wise application of all filters removed dive fragments that did not comply with the applied criterion; a small percentage of drift fragments remained. Each colour corresponds to the effect of applying one filter. If a filter was applied, the corresponding shaded section was removed.

Filtering the data by step-wise addition of selection criteria decreased the fraction of dive fragments that were retained (Fig. 3). The percentage of dive fragments that were discarded by application of each selection criterion (Table 3) and the total number of drift fragments identified for each seal depended on their individual diving behaviour.

**Table 3.**
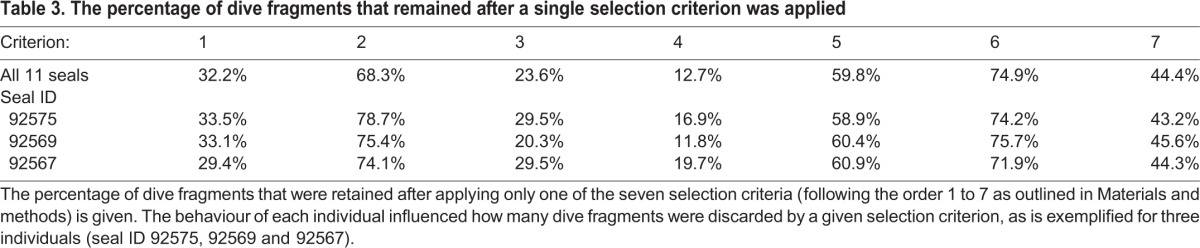
**The percentage of dive fragments that remained after a single selection criterion was applied**

The frequency distribution of the filtered data was non-normal or skewed for most dive variables. Hence the median and inter-quartile range (IQR) are reported as summary statistics.

During the 2322 drift fragments, seals descended whilst drifting with an average drift rate of −0.17 m s^−1^ (IQR: −0.26 to −0.10 m s^−1^). Within the range of the relevant selection criterion, the selected drift fragments, during which seals descended, were terminated at depths of 391.3 m (IQR: 441.3 to 336.3 m). Most drift fragments lasted for 811.8 s (IQR: 637.6 to 1037.3 s) and drift fragments comprised 51.6% of the total dive duration (IQR: 45.2% to 58.1%). The highest occurrence of drift fragments was between 05:00 lt and 06:00 lt.

**Fig. 4.**
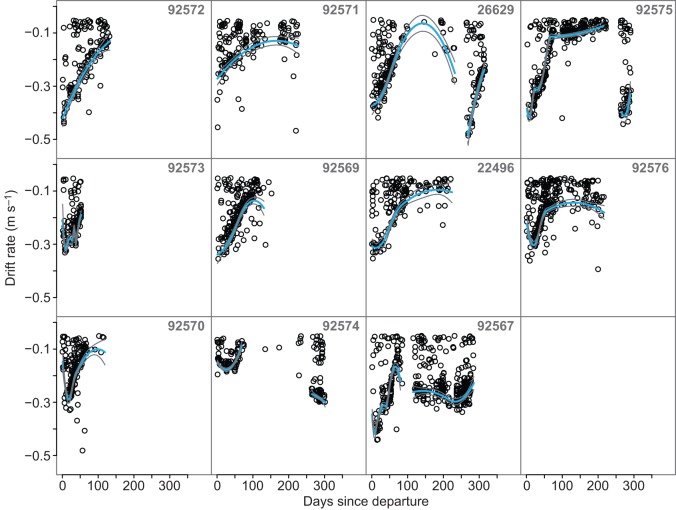
**Time traces of extracted drift rates for each individual.** Each black circle represents a drift rate of a selected drift fragment, while the blue lines represent constrained beta splines (cobs) fitted with confidence intervals in grey (see Materials and methods). For three individuals (seal ID 26629, 92575 and 92574), parts of the post-breeding migrations were also recorded. The buoyancy of these individuals drastically decreased post-breeding.

The time series of drift rates (Fig. 4) display strong fluctuations in buoyancy for each individual. At the beginning of the post-moult migration, the drift rate of most individuals was between −0.3 and −0.4 m s^−1^. Within 75–150 days of departure from South Georgia, all individuals reached a peak in buoyancy. Thereafter, their buoyancy fluctuated around near-neutral buoyancy until hauling out. For three female individuals (ID 26629, 92575 and 92574), the extracted drift rate time series included both the post-moult and part of the post-lactation migration. Comparison of the number of drift dives identified in the first 28 days of each respective migration revealed that seal 26629 performed significantly more drift dives during the post-moult (*N*=56) than during the post-lactation migration (*N*=36; χ^2^=4.348, d.f.=1, *P*=0.037). Contrarily, seal 92574 performed more drift dives during the post-lactation migration (*N*=65) than during the post-moult (*N*=39; χ^2^=6.5, d.f.=1, *P*=0.011). While during the post-lactation migration of seal 92575 more drift dives were identified (*N*=52) than during the post-moult migration (*N*=37), the difference was not statistically significant (χ^2^=2.528, d.f.=1, *P*=0.112).

### Independent verification

**Fig. 5.**
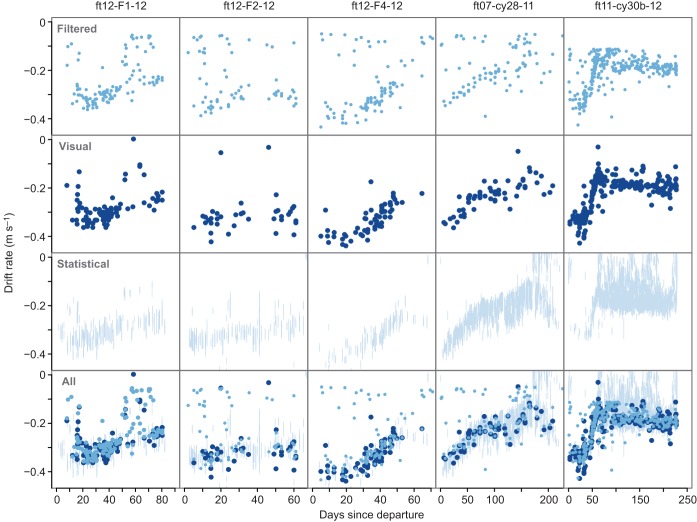
**Time traces of drift rates used for independent verification.** Each column displays for each individual four corresponding drift rate time series. In the ‘filtered’ row, the drift rate time series illustrated by light blue points were obtained with the step-wise filtering method. Drift rates that were visually confirmed and measured using detailed high-resolution data are displayed by dark blue points in the row labelled ‘visual’. In the ‘statistical’ row, light blue lines represent drift rates obtained with a statistical detection algorithm adapted from Dragon et al. (2012) (see Materials and methods). The last row displays the drift rates obtained by all three methods together, where light blue points depict the filtered time series, dark blue points represent the visual time series and light blue lines show the statistical time series. Strong coherence in the filtered and visual time series is apparent for every individual and the trends over time are consistent with those of the unsmoothed statistical time series. This is further illustrated by the overlap of points and lines in the combined time series.

For seals from the Kerguelen deployments, the filtering method selected between 2.2% and 3.9% of an individual's broken-stick abstracted dive fragments as drift fragments. Between 60% and 81% of these selected drift fragments could be visually confirmed as being true drift fragments (Table 4). The manually re-measured drift rates displayed similar daily variation to the drift rates extracted from broken-stick abstracted dive fragments (Fig. 5). The time series obtained with the statistical algorithm presented more noise than the other two methods. Yet, aside from the noise, the observed temporal patterns in each individual's set of drift rate time series were overall coherent. Further, the drift rate time series obtained with the filtering method and the visually confirmed time series were all significantly positively correlated (Table 1).

**Table 4.**
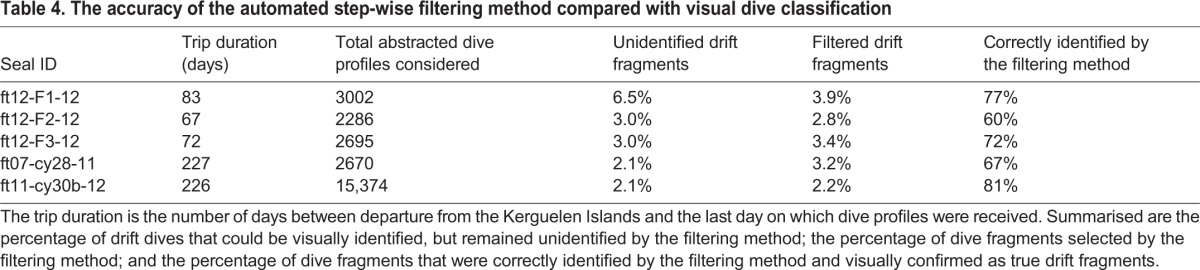
**The accuracy of the automated step-wise filtering method compared with visual dive classification**

## DISCUSSION

In this study, an improved knowledge-based method for detecting buoyancy changes was demonstrated by filtering abstracted dive profiles of 11 elephant seals from South Georgia for drift fragments. The step-wise filtering method was independently verified using detailed high-resolution data of five additional elephant seals from the Kerguelen Islands for which the contemporaneous abstracted data telemetered via Argos were available. The selected drift fragments rendered time series of drift rates that displayed marked buoyancy changes during the migrations. The fitted splines further emphasise the trends in buoyancy change.

The broken-stick abstraction of detailed dive data enables persistent transmission of long time series via telemetry, which captures more variation than short high-resolution time series. Whilst for the detection of other behavioural modes, such as foraging, less abstraction and more broken-stick points may be required (Heerah et al., 2014), the minimum of four inflection points sufficiently conveys the geometric shape (Fedak et al., 2001) to depict enough detail for abstracted drift dive profiles to be adequately represented and identifiable. Despite the reduced resolution of the abstracted dive data, the time series obtained with the filtering method depict the buoyancy changes almost as well as the detailed high-resolution time series, indicating that the broken-stick algorithm is able to accurately abstract drift dives because of the low variability in change in depth of the bottom phase (Photopoulou et al., 2015).

Nevertheless, there was noise in the drift rate time series, which possibly resulted from inexact time and depth recordings after the compression was applied, which propagated through the data upon calculating the vertical speeds. Data binning and compression before satellite transmission of the data could also have caused noise. Additionally, the broken-stick algorithm is unable to capture instantaneous changes in vertical speed. Therefore, parts of the deceleration and acceleration phases immediately before or after the drift phase are often included in the abstracted drift fragment, leading to an over- or under-estimation of the true drift rate.

The detailed high-resolution time series obtained with the statistical method appeared to be noisier than the other two methods. This is probably due to the fact that the statistical method did not identify single drift fragments like the other two methods, but rather took multiple measurements of the mean drift rate in an 8 min sliding window. The time series of ft07-cy28-11 and ft11-cy30b-12 in particular were noisier because the standard deviation parameters had to be adjusted in order to obtain a complete drift rate time series (see Materials and methods). Overall, the noise might have been reduced by applying a smoothing filter; however, considering that the high-resolution time series were solely used for visual comparison, it was deemed unnecessary.

The five sets of drift rate time series used for the independent verification displayed remarkable coherence in the apparent trends in buoyancy change. The detailed high-resolution drift rate time series revealed how much dive information is inaccessible for satellite-received time series because of the intermittent transmission of limited random samples. The positive correlations between the filtered and visually inspected time series manifest that – despite detection errors – the step-wise filtering method reliably detects changes in buoyancy. These positive correlations would be even stronger if only the beginnings of the corresponding time series, during which the buoyancy changes are most pronounced, were compared.

Visual inspection of the detailed high-resolution data with the corresponding satellite-received abstracted dive profiles revealed that the step-wise filtering method is most prone to type I errors. However, a large majority of the unidentified drift dives occurred at shallow depths, were short in duration or included multiple short drift fragments per dive that could not be adequately represented in the abstracted dive profile. Thus, the drift rates of these unidentified drift fragments would probably be inaccurate as a result of residual air in the lungs or false abstraction of the drift fragment and therefore would be inutile for further analysis, even if they had been correctly identified.

Failure of the abstraction algorithm to convey the geometric shape using four at-depth inflection points was the most common source of type II errors. In particular, dive profiles with oscillating bottom wiggles, also termed D dives (Crocker et al., 1997; Le Boeuf et al., 1992), were often falsely represented by the abstracted dive profile. This is because the broken-stick algorithm is limited to two inflection points to describe the geometric shape of the bottom phase, which is insufficient to adequately capture the activity in the bottom phase (Photopoulou et al., 2015). The abstracted profiles of such dives are thus similar in appearance to drift dive profiles and can only be discerned in the high-resolution data. Dives of type D have also been most commonly misclassified as drift dives by the random forest identification method (Thums et al., 2008a) and we confirm that seals appear to drift on parts of the depth oscillations at the bottom of these dives, which is the likely reason for misclassification also in abstracted dives.

While type II errors of the step-wise filtering method seemingly can only be reduced by visual inspection of the high-resolution dive profiles, type I errors could be decreased by refining or adding selection criteria informed by future studies of drift diving behaviour. Refinements may be required because the behaviour of an individual seal influences how its body condition improves. For example, it is rare for females to reach positive buoyancy during post-lactation migrations (Thums et al., 2008a), so the step-wise filtering method excludes positive drift fragments *a priori*. However, if positive buoyancy is likely to be expected, the filtering method should be adapted to accommodate this information. The random variability caused by individual behaviour can otherwise be a potential source of error (Photopolou, 2012), because the effectiveness and relevance of each selection criterion depends on an individual's behaviour.

Variation in drift dive profiles as a result of both individual behaviour and an individual's location is well illustrated by the daily variation of drift rates, which is apparent in all drift rate time series regardless of the method used to obtain them. The proportion of drift fragments selected by the filtering method was small, which supports previous findings that drift dives only constitute small parts of the dive records (Crocker et al., 1997; Dragon et al., 2012; Le Boeuf et al., 2000). The visual inspection of detailed high-resolution data further established that compared with other dive types the proportion of drift dives was consistently small, but the physical amount of performed drift dives varied up to threefold among individuals. Whilst in this study, differences in the number of drift dives between the post-moult and post-lactation periods varied inconsistently depending on the individual, post-moult and post-lactation differences as found by Thums et al. (2008b) seem likely. The lack of a consistent relationship in our study is probably due to the small number of individuals for which both post-moult and post-lactation periods could be recorded. Overall, the variation in the amount of drift dives among and within individuals poses interesting questions regarding the purpose of drift diving. For example, if drift dives serve as resting and food-processing dives, as has been suggested (Crocker et al., 1997; Mitani et al., 2010), does the variation in the amount of drift dives indicate varying needs for resting or food processing? Or are there other ways of resting, e.g. by extending surface intervals? Further, are gaps in the drift rate time series a result of a reduced need for drifting or due to a failure of the methods to detect drift dives?

The latter is particularly important because the quadratic relationship between drag and velocity can lead to discontinuities in the drift dive record around neutral buoyancy (Biuw et al., 2003). To overcome such discontinuities, the data are extrapolated for example by fitting splines. Thus, the reason for such discontinuities has implications for the interpretation of the resulting fluctuations in drift rate around neutral buoyancy. Generally, any such fluctuations should be interpreted cautiously with respect to body condition or dive behaviour. This is particularly important in phases of seemingly constant buoyancy, during which fluctuations could also result from slight changes in the ratio of lean to lipid tissue together with a change in total body mass.

Individual dive behaviour can affect the detectability of buoyancy changes. This was especially apparent for the two individuals 26629 and 92567. The drift rate time series of these individuals displayed a cluster of very slow or even positive vertical speeds during early parts of the migration when such vertical speeds are highly unlikely. Such a cluster of slow vertical speeds was also detected in the time series of ft11-cy30b-12 from the Kerguelen deployment. The dive fragments from which these vertical speeds stem have similar characteristics to drift fragments and therefore could not be filtered out by any of the stated selection criteria. This provides evidence for the presence of a second behaviour other than drift diving, which is depicted similarly by the two-dimensional dive profiles in that the dive fragments have low variability in depth change over extended periods of time. The two-dimensional representation of the dive data may mask a behavioural movement in the third dimension which could, for example, be associated with more complex dive patterns. Taking note of the fact that such clusters appear whilst these seals forage in the area of the Bullard Fracture Zone and Bruce Ridge, South Atlantic, and the Kerguelen Plateau, southern Indian Ocean, such complex dive patterns could probably arise in response to the complex topography that can, for example, induce changes in the water column by upwelling.

In the Kerguelen deployment, such clusters were only visible in the time series obtained with the step-wise filtering method and the statistical method, but not in the visually confirmed time series. This illustrates that both automated drift dive identification methods were unable to distinguish between such dive fragments and true drift fragments, whilst subjective visual inspection of detailed high-resolution data enabled the two to be differentiated. However, the broken-stick abstracted dive profiles of these other dive fragments are indiscernible from true drift dive fragments. Thus, with broken-stick data alone, these clusters currently can only be eliminated by manually removing vertical speeds that are inconsistent with the apparent temporal patterns in the drift rate time series. Presently, a reliable distinction between these dive fragments and true drift fragments only seems possible with acceleration data and further investigations will be needed to ascertain the form and function of the aforementioned second behaviour.

Generally speaking, if any additional or alternative information on an individual's or another species' diving behaviour is available, this information can be accommodated by simply adjusting the selection criteria. For example, Andersen et al. (2014) used a preliminary version of our method (Gordine, 2013) and successfully identified drift dives in the dive records of hooded seals (*Cystophora cristata*). The selection criteria and the spline-fitting algorithm presented here are by no means unchangeable or finite and need to be fine-tuned to a species or an individual. Other statistical methods such as the random forest algorithms require additional datasets for training the algorithm, which can be problematic for small datasets and which also requires time-consuming visual classification. The advantages of our method are that it is fast, automated and standardised. Its implementation is easy for small and large datasets and the results are reproducible. This enables utilisation of our step-wise filtering method as a first step in drift dive analysis, after which further individual refinements can be made.

Given the abstracted nature of any time–depth record, any drift dive identification method is prone to error, be they visual dive classification or random forest algorithms trained by visually classified datasets (Thums et al., 2008a). The important question is whether a given method is able to convey the relevant information despite its inherent noise or error. Our method provides long drift rate time series from abstracted dive profiles that, despite its error rate, captures the relevant variations in buoyancy change over a migration. While new methodologies based on on-board processing are increasingly developed and used, this approach could substantially increase the amount of information about the well-being of marine top predators by accessing older, archived data of abstracted nature. Together with newly collected high-resolution data, valuable long time series are made available to address and monitor changes in the physical environment and body condition.
